# Travel-associated Legionnaires’ disease: would changing cluster definition lead to the prevention of a larger number of cases?

**DOI:** 10.1017/S0950268818003266

**Published:** 2018-12-03

**Authors:** M. C. Rota, A. Bella, M. G. Caporali, A. Nicolau, V. Drasar, M. L. Ricci, M. Scaturro, M. Gumá, S. Crespi

**Affiliations:** 1 Dipartimento di Malattie Infettive, Istituto Superiore di Sanità, Rome, Italy; 2 Conselleria de Salut i Consum, Palma de Mallorca, Spain; 3 Public Health Institute Ostrava, Vyskov, Czech Republic; 4 Biolinea Int., Palma de Mallorca, Spain

**Keywords:** Accommodation site, Legionnaires' disease cluster, TALD

## Abstract

According to European Guidelines for Legionnaires’ Disease prevention and control, travel-associated Legionnaires’ disease (TALD) cases are managed differently if classified as sporadic or as part of a cluster and more stringent control measures are deployed after clusters are identified. In this study, we propose to modify the current cluster definition: ‘two or more cases of Legionnaires’ disease (LD) who stayed at, or visited, the same commercial accommodation site 2–10 days before onset of illness and whose onset is within the same 2-year period’ with a new cluster definition, i.e. accommodation sites associated with multiple cases regardless of the time elapsed between them. TALD cases occurred in Italy and in the Balearic Islands between 2005 and 2015 were analysed applying the current European Legionnaires’ Disease Surveillance Network (ELDSNet) cluster definition. In a sample of selected accommodation sites with multiple cases, a microbiological study was also conducted. Using the new definition, 63 additional sites (16.4% increase) and 225 additional linked cases (19.5% increase) were identified. *Legionella pneumophila* sg1 was isolated from 90.7% of the selected accommodation sites. The use of the here proposed TALD cluster definition would warrant a full investigation for each new identified case. This approach should therefore increase the number of sites that will require a risk assessment and, in the presence of an increased risk, the adoption of LD control measures to hopefully prevent additional cases.

## Introduction

In 1987, the European Surveillance Scheme for travel-associated Legionnaires’ disease (TALD), later called EWGLINET, was firstly introduced [1] and since April 2010, the scheme is called European Legionnaires’ Disease Surveillance Network (ELDSNet) and it is coordinated by the European Centre for Disease Prevention and Control (ECDC) based in Stockholm, Sweden [2].

Legionnaires’ disease (LD) cluster definition changed over time. In particular, until 2001, a cluster of TALD was defined as two or more LD cases who stayed at the same accommodation site during the incubation period and whose onset was within a 6-month period, while cases occurring at sites with previous cases more than 6 months earlier were categorised as ‘linked’. A change in cluster definition was made at the beginning of 2001 and ratified in 2002, by the European Guidelines for LD prevention and control [3]. According to the new definition, a cluster was defined as two or more cases of LD who stayed at or visited the same commercial accommodation site in the 2–10 days before onset of illness and whose onset was within the same 2-year period. This change in the definition of clusters brought to a rise in the number of clusters detected from 28 in 2000 to 72 in 2001, of which 43 (60%) would have met the old cluster definition, with a gain of 29 extra clusters. In fact, many of the linked cases that the previous definition would have identified were absorbed into these ‘new’ clusters [4].

Accommodation sites associated with TALD clusters investigated by the Network that were associated with at least one additional case in a 2-year period, were defined as ‘reoffending sites’ [5].

All cases occurring over 2 years before the initial case or 2 years after the latest case in a cluster are considered sporadic by the present cluster definition. This has important consequences, as TALD cases are managed differently if classified as sporadic or as part of a cluster and more stringent control measures are deployed after clusters are identified. Identification of a cluster warrants immediate action by the ELDSNet coordinating centre and the public-health authorities in the involved country, while for sporadic cases public health authorities have only to ensure that the notified site receives the checklist outlining good practice for minimising the risk of *Legionella* infection.

We propose to modify the cluster definition to consider as part of a cluster any new TALD case identified in a known accommodation site, regardless of the time elapsed from the latest identified case. The underlying idea is that all TALD with an epidemiological link to the same accommodation site should be managed, in terms of infection control purposes, accordingly to European LD cluster guidelines.

In this study, TALD data collected between 2005 and 2015 in Italy and Balearic Islands, two prominent touristic destinations in Europe for which TALD datasets were available to the authors, were analysed using the current ELDSNet cluster definition and the newly proposed cluster definition. In a sample of selected accommodation sites associated with multiple cases and at least one positive environmental microbiological finding an analysis of the characteristics of *Legionella* strains isolated was also conducted.

The impact on public-health interventions of the two definitions is discussed.

## Methods

LD is a statutorily notifiable disease in all EU/EEA Member States and individual cases of TALD are reported by the nominated network members to the ELDSNet coordinating centre in Stockholm.

According to the ELDSNet definition a TALD case is a case who in the 2–10 days before onset of illness stayed at or visited a commercial accommodation site that has not been associated with other cases of LD in the 2 years prior to date of onset of illness. A cluster is defined as two or more cases who stayed at or visited the same commercial accommodation site in the 2–10 days before onset of illness and whose onset is within the same 2-year period (Table 1).

**Table 1.** European definitions for single case, cluster and re-offending site and newly proposed cluster definition

Single-case definition	Cluster definition	Reoffending sites	Newly proposed cluster definition
According to ELDSNet			
Cases who in the 2–10 days before onset of illness stayed at or visited a commercial accommodation site that has not been associated with any other cases of LD, or cases who stayed at an accommodation site linked to other cases of LD more than 2 years previously	Two or more cases who stayed at or visited the same commercial accommodation site in the 2–10 days before onset of illness and whose onset is within the same 2-year period	Sites associated with clusters investigated by the Network that were associated with at least one further case in a 2-year period	Sites associated with at least two cases regardless of the time elapsed between them

TALD cases are to be reported as soon as they are laboratory confirmed and travel information is obtained (accommodation address, telephone number, web page URL, room number and any recognised exposure risks).

Once a case is reported to the ECDC database TESSy, accommodation details are checked in order to identify possible previously reported TALD cases associated with the same accommodation site. If no such cases are identified in the previous 2 years, the accommodation site is classified as ‘single-site notification’.

### Epidemiological study

All TALD cases diagnosed in Europe between 2005 and 2015 according to European case definition, who travelled to Italy and to the Balearic Islands, were analysed applying the current ELDSNet single case and cluster definitions, and a new here proposed cluster definition.

The datasets included residents of Italy and Balearic Islands, who travelled in Italy and in the Balearic Islands respectively, and all non-Italian and Balearic residents who travelled to Italy or to Balearic Islands and stayed in accommodation sites in one of the two countries. Italian and Balearic Islands residents who acquired LD in accommodation sites outside Italy or Balearic Islands were excluded from the two datasets.

The here proposed new cluster definition includes sites associated with at least two cases regardless of the time elapsed between them, and therefore not only those sites with sporadic cases which do not qualify as clusters under the current definition, but also any other possible combination of sporadic cases and clusters during the entire study period. According to this definition, only the initial case is considered sporadic whilst all the subsequent ones are considered a possible recurrence and therefore part of the same cluster.

### Microbiological assessment

In order to assess the characteristics of Legionella strains collected from accommodation sites associated with LD clusters, we conducted an in depth analysis on environmental samples collected from 54 accommodation sites. Accommodation sites were selected for this in depth analysis if associated with at least one cluster, one additional case and at least one positive environmental microbiological finding. These included accommodation sites in the mentioned dataset as well as sites investigated by co-authors in other countries mainly in Spain and in the Czech Republic.

Microbiological investigation of environmental samples was performed according to the European guidelines [3].

Where available, environmental isolates were characterised using monoclonal-antibody (MAb) typing according to Dresden panel [6] and sequence-base typing (SBT) (http://www.hpabioinformatics.org.uk/legionella/legionellasbt/php/sbthomepage.php).

Data were analysed by STATA 11.2 software (Stata Corporation, College Station, Texas, USA).

## Results

We analysed 2921 TALD cases, of which 2686 travelled in Italy and 235 in the Balearic Islands. According to the current case definition, in Italy, single cases were 1616 (60.1%), while the remaining 1070 (39.9%) cases were associated with clusters. In the Balearic Islands single cases reported were 154 (65.5%), and cluster cases were 81 (34.5%). Overall the 2921 cases stayed in 1982 accommodation sites of which 1813 located in Italy and 169 in Balearic Islands. The total number of cluster-associated sites identified in the study period was 385 with 1151 associated cases.

The re-offending sites (i.e. accommodation sites linked to clusters and associated with at least one further case in a 2-year period) represented 23.5% of the total number of cluster in Italy and 18.5% in Balearic Islands.

**Fig. 1.**
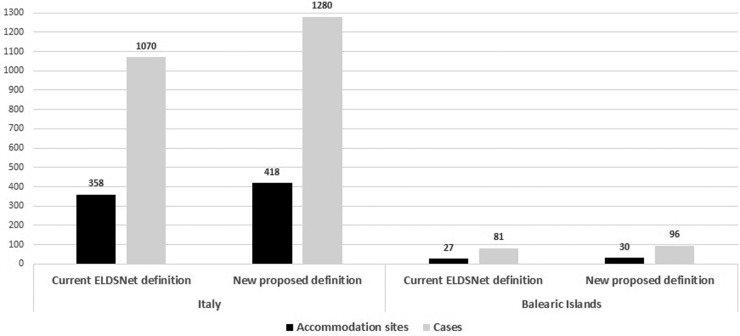
Number of accommodation sites and cases associated with clusters according the current ELDSNet cluster definition and to the new proposed definition.

Using the newly proposed definition, 63 additional sites (16.4% increase) and 225 additional linked cases (19.5% increase) were identified (60 sites and 210 cases in Italy and 3 sites and 15 cases in Balearic Islands) (Table 2, Fig. 1). The average time interval between the first and the second case was approximately 4 years with the second case occurring within 3–4 years from the first case in 55% and from 5 to 9 years in 25% of the accommodation sites.

**Table 2.** TALD cases and accommodation sites reported in Italy and Balearic Islands in 2005–2015

Definition		Italy, *N* (%)	Balearic Islands, *N* (%)	Total *N* (%)
Current ELDSNet definition	TALD cases	2686	235	2921
	Number of accommodation sites	1813	169	1982
	Single cases	1616/2686 (60.1)	154/235 (65.5)	1770/2921 (60.6)
	Cluster sites	358	27	385
	Cluster cases	1070/2686 (39.9)	81/235 (34.5)	1151/2921 (39.4)
	Reoffending sites^a^	84/358 (23.5)	5/27 (18.5)	89/385 (23.1)
	Cases associated with reoffending sites	345/1070 (32.2)	8/81 (9.8)	353/1151 (30.6)
New proposed cluster definition	Cluster sites	418	30	448
	Cluster cases	1280	96	1376

a According to the definition in Ricketts *et al*. (2010).

As for the 54 accommodation sites (mostly hotels) associated with recurrent cases, included in the microbiological assessment of this study, they were associated with a total of 419 TALD cases (an average of seven cases per site, range 3–50).

A wide range of *Legionella* counts was observed in environmental samples: from sites with a single sample positive at a concentration <10^3^ CFU/l to sites with the majority of positive samples ranging from low to extremely high counts (range: 10^2^–10^6^). *Legionella pneumophila* sg1 was isolated from 90.7% (49/54) of the above accommodation sites. MAb typing data were available for 32 sites, 81.2% (26/32) of which reacted with MAb 3/1 from the Dresden panel. SBT typing data were available for 21 sites; sequence type (ST) 1 found in six sites was the most frequently identified. Eighteen clinical strains were available from patients who stayed in 13 accommodation sites and *L. pneumophila* sg1 clinical and environmental strains resulted indistinguishable by ST type in 10 sites (Table 3).

**Table 3.** Typing of environmental and clinical strains isolated from 54 accommodation sites with recurrent cases in 2005–2015

Site	*Legionella* spp.	Serogroup	Mab 3/1	ST (environmental)	ST (clinical)	
1	*L. pneumophila*	6	NA	NA	NA	
2	*L. pneumophila*	1	Pos	42	NA	
3	*L. pneumophila*	1, 5, 8	Neg	1	NA	
4	*L. pneumophila*	1	Pos	1584	NA	
5	*L. pneumophila*	1	NA	NA	NA	
6	*L. pneumophila*	1	NA	NA	NA	
7	*L. pneumophila*	1	Pos	NA	NA	
8	*L. pneumophila*	1, 6	Neg	1	NA	
9	*L. pneumophila*	1, 6	Pos	NA	NA	
10	*L. pneumophila*	1	Pos	NA	NA	
11	*L. pneumophila*	1	Pos	NA	NA	
12	*L. pneumophila*	1	NA	NA	NA	
13	*L. pneumophila*	1, 5	Neg	1	NA	
14	*L. pneumophila*	1	Pos	23	NA	
15	*L. pneumophila*	1	Pos	42	42	
16	*L. pneumophila*	1	Pos	885	885	
17	*L. pneumophila*	1	Pos	23	23	
18	*L. pneumophila*	1, 5, 3	Neg	NA	47, 146	
19	*L. pneumophila*	1, 6	Pos	Novel	NA	
20	*Legionella* spp.		NA	NA	NA	
21	*L. pneumophila*	1	Pos	616	616	
22	*L. pneumophila*	1	NA	NA	NA	
23	*L. pneumophila*	1	Pos	42, 1	42	
24	*L. pneumophila*	1, 3	Neg	NA	NA	
25	*L. pneumophila*	1	Pos	1	1 (×2)^a^	
26	*L. pneumophila*	1	Pos	1090	1090 (×2)^a^	
27	*L. pneumophila*	1	Pos	20	20(×2)^a^	
28	*L. pneumophila*	1, 6	Pos	146, 2156, 435	2156, 435	
29	*L. pneumophila*	1, 5	Neg	NA	NA	
30	*L. pneumophila*	1	NA	NA	NA	
31	*L. pneumophila*	1	NA	NA	NA	
32	*L. pneumophila*	1	NA	NA	NA	
33	*L. pneumophila*	1	Pos	1	NA	
34	*L. pneumophila*	1	NA	NA	NA	
35	*L. pneumophila*	1, 6	NA	NA	NA	
36	*L. pneumophila*	1	NA	NA	Clinical strain available, SBT not performed	
37	*L. pneumophila*	1	Pos	NA	NA	
38	*L. pneumophila*	2–14	Pos	NA	NA	
39	*Legionella* spp.		NA	NA	NA	
40	*L. pneumophila*	1, 8, 10	Pos	NA	NA	
41	*L. pneumophila*	1	NA	NA	NA	
42	*L. pneumophila*	1	NA	NA	NA	
43	*L. pneumophila*	1	NA	NA	NA	
44	*L. pneumophila*	1	Pos	23	23	
45	*Legionella* spp.		NA	NA	NA	
46	*L. pneumophila*	1	NA	NA	NA	
47	*L. pneumophila*	1	NA	NA	NA	
48	*L. pneumophila*	1	Pos	NA	NA	
49	*L. pneumophila*	1	Pos	NA	NA	
50	*L. pneumophila*	1	NA	NA	NA	
51	*L. pneumophila*	1	NA	NA	23	
52	*L. pneumophila*	1	Pos	901	NA	
53	*L. pneumophila*	1	Pos	901	NA	
54	*L. pneumophila*	1	Pos	182	NA	

a (×2) means that two undistinguishable strains were isolated in different years in the same accommodation site.

## Discussion

According to the ECDC, between 2010 and 2015, a yearly average of approximately 900 TALD cases was reported in Europe, with a 20% increase observed between 2014 and 2015. Approximately 30% of these cases were part of clusters, outbreaks or cases associated with the same site. Italy is among the countries reporting the highest number of TALD cases in Europe [7], while the Balearic Islands report few cases every year. Interestingly, despite the different magnitude, the two datasets are quite similar in terms of percentages and both are in line with European data: about 60–65% of sporadic cases, 35–40% of cluster cases and 18–23% of reoffending sites. The only relevant difference is that accommodation sites associated with recurrent cases in the Balearic Islands are linked to fewer cases than the ones in Italy. This difference may be affected by differences in the size and demographic characteristics of the visiting populations.

Our findings suggest that, compared with the ELDSNet definition, the newly proposed cluster definition would allow the identification of an increased number of cluster cases (+19.5%) and, consequently, of accommodation sites associated with clusters (+16.4%). This, in turn, would enable a more thorough investigation (including a compulsory environmental investigation) of the additional cluster-associated accommodation sites as required by European guidelines.

*L. pneumophila* sg1 Mab 3/1 positive strains are responsible for the majority of LD cases and therefore considered more virulent: an increased hydrophobic cell envelope is thought to allow enhanced survival in aerosols, hence increased capacity to cause infection [6, 8, 9]. The high prevalence (81.2%) of MAb 3/1 positive strains among environmental isolates in our dataset, strongly suggests that this is a critical feature of recurrent sites and highlights the importance of MAb typing. In four sites with recurrent cases, MAb 3/1 positive strains were repeatedly isolated in different years, in one site in particular even 12 years after the initial finding. In three out of four sites (75%), the environmental strains were indistinguishable from the clinical strains.

Unfortunately, due to the limited use of culture for LD diagnosis in Europe and the low *Legionella* isolation rate when culture is used [8], we were able to compare clinical and environmental strains only for 10/54 (18.5%) accommodation sites.

Although related to a time frame different from this study, it is worth mentioning that indistinguishable *L. pneumophila* strains (sg1, MAb3/1 positive, ST901) were responsible for two cases who stayed in the same hotel 24 years apart from each other, namely 1987 and 2011 (author personal communication). These findings suggest that a 2-year cut off does not in principle exclude the possibility of persisting colonisation of accommodation sites leading to possible new cases of infection.

Findings of our study are very different from those obtained from routine sampling, conducted in the absence of cases reported in the literature [10–15]. In fact, in routine samples only 20–45% of the sites are colonised by *Legionella pneumophila* sg1, and of these only 6–15% are MAb 3/1 positive.

Outcome of our study could have been potentially biased by the fact that only recurrent sites with positive environmental cultures were included in the analysis. However, more than 60% of our accommodation sites associated with clusters are positive at the first investigation and this percentage increases further after repeated investigations.

By contrast, *Legionella* counts in positive samples collected in sites with recurrent cases are not very different from those obtained by random sampling in sites not associated with TALD cases.

Prospective studies targeted at demonstrating the correlation between environmental and clinical strains linked to the same accommodation sites for periods longer than 2 years, would be useful to confirm our observations.

The new ELDSNet Operating Procedures [16] state that when a single TALD case is identified the notification should include also the number of previous cases reported within the last 5 years. However, even though in the previous 5 years the hotel was associated with LD cases, if more than 2 years passed from the latest case, the ELDSNet contact point in the country of infection is only required to ensure that the relevant accommodation site receives the checklist that outlines good practice for minimising the risk of *Legionella* infection. On the contrary, if the here proposed definition (i.e. accommodation sites with more than one case over 5 years, regardless of when the cases occurred as clusters) is applied, a thorough investigation should be required. This change in the procedure would have a great impact on prevention of LD cases, since public-health authorities would actively investigate also those accommodation sites associated with multiple cases that are not included under the current cluster definition and, where needed, additional control measures could be recommended, preventing the occurrence of further cases. In fact, investigations that include sampling are expected for all cluster sites, and they have shown a number of accommodation sites to be positive for *Legionella*. Whilst these sites cannot conclusively be proven as the source of infection in the absence of clinical isolates for comparison, the presence of *legionella* is highly suggestive of the sites being the source. Moreover, the new definition may identify more re-offending sites and this will allow for more studies to be carried out that may help determine why some sites continue to harbour the organism despite disinfection and control measures.

To establish the cost/benefit ratio of this newly proposed definition is not the purpose of this study, but further studies in this direction would be useful. It would be appropriate to estimate whether the costs of : enhanced surveillance for both, the coordination centre and the ELDSNet collaborators in the countries; the greater number of inspections of the accommodation site; laboratory tests to be dealt with by the local health authorities; and to be faced by tour operators or hoteliers in implementing control measures and in the case of withdrawal of customers in the presence of large clusters; are justified by the gain in terms of individual and public health.

### Limitations of the study

The microbiological assessment was conducted on a convenience sample of accommodation sites that included sites in Italy, Balearic Islands as well as other countries. The reason for this choice was that data from recurrent accommodation sites in Italy and the Balearic Islands alone was limited. To make the analysis more robust, information collected during environmental investigations by co-authors in accommodation sites with recurrent cases in other countries, mainly Spain and the Czech Republic, were also included. As only sites for which the required data for this analysis was available were selected for this assessment, the results might not give a complete picture of all recurrent sites.

Further, since ELDSNet operative procedures currently in use do not oblige to carry out a full environmental investigation in the presence of a single case, data on non-recurring sites were too limited to allow a comparative assessment. For this reason, microbiological data from routine samplings reported in the literature were used as a comparison.
